# Transport through a correlated polar side-coupled quantum dot transistor in the presence of a magnetic field and dissipation

**DOI:** 10.1038/s41598-023-51142-9

**Published:** 2024-01-10

**Authors:** Hemant Kumar Sharma, Manasa Kalla, Ashok Chatterjee

**Affiliations:** 1 Institute of Physics, Bhubaneswar, Odisha India; 2 National Institute of Science Education and Research, Bhabaneswar, Odisha India; 3 Department of Physics, GITAM School of Sciences, GITAM University, Rudraram, Hyderabad, Telangana India

**Keywords:** Materials science, Nanoscience and technology, Physics

## Abstract

Non-equilibrium magneto-transport properties of a quantum dot dimer transistor are studied in the presence of electron–electron and electron–phonon interactions and the interaction of the dimer phonons with the substrate phonon bath that gives rise to dissipation. The entire system is modeled by the Anderson–Holstein–Caldeira–Leggett Hamiltonian where the Caldeira–Leggett term takes care of the damping. The electron–phonon interaction is dealt with the Lang–Firsov transformation and the electron–electron interaction is treated at the mean-field level. The transport problem is studied using the Keldysh non-equilibrium Green function theory and the effects of electron–electron interaction, external magnetic field, electron–phonon interaction and damping on spectral function, tunneling current and differential conductance of the dimer transistor are calculated.

## Introduction

The study of transport through nanoscale systems like a single molecular transistor (SMT) has been the subject of extensive investigations in recent years^1–10^. A single molecular transistor contains in its central region a molecule or a quantum dot (QD) or any nanosystem that has discrete energy levels. The tunneling current flows through the SMT device because of an applied bias voltage and the current can be controlled by a gate voltage^11–13^. The first SMT device was build using *C*_60_^14,15^. Some of the interesting phenomena such as Kondo effect^16–19^, Fano effect^20–22^, Coulomb blockade^23,24^, Dicke effect^25,26^ and Josephson tunneling^27–29^ etc. have been observed in SMT systems at low temperature. The electron–phonon interaction gives rise to a polaronic effect^30–37^ in polar semiconductor QDs and consequently the quasi particles that take part in transport phenomena in an SMT device with a central polar QD are naturally polarons which are electrons dressed with virtual phonons. Thus, in general, the transport properties in an SMT device are considerably influenced by both electron–electron (e–e) and electron–phonon (e–p) interactions. Chen et al.^38^ have shown that the e–p interaction sharpens the zero-phonon peaks and produces side bands in the spectral density. They have also investigated the dependence of the chemical potential of leads on various transport properties at zero temperature. The quantum transport in the SMT devices have been studied by various theoretical and numerical methods like rate equation approach^39^, kinetic equation method^40,41^, non-crossing approximation method^42^, slave-boson mean-field method^43^, numerical renormalization^44–48^ and non-equilibrium Green’s function approaches^49–52^.

The effect of quantum dissipation on quantum transport has been recently investigated by Raju and Chatterjee (RC)^53^ RC have considered an SMT system placed on an insulating substrate that contains a collection of decoupled harmonic oscillators. The substrate can act like a phonon bath and interact with the phonon mode of the SMT giving rise to a dissipative effect. Costi^54^ has studied an SMT device in the presence of an external magnetic field using Wilson’s numerical renormalization group technique and suggested that a strongly coupled QD can act as a spin filter in a magnetic field. Dong et al.^55^ have shown that a magnetic field can suppress the linear conductance at zero temperature and give rise to side peaks at a sufficiently increased value. Chatterjee and collaborators^56^ have examined the effect of damping on the magneto-transport properties in SMT and also the effect of temperature on the tunneling current in an dissipative SMT system. Chatterjee and collaborators have recently studied the transient dynamics of an SMT device^57^.

Several studies have also been made on the molecular transistor systems that are extensions of the SMT device. These systems typically involve two or more QDs in the central region. Among them, the bimolecular transistor (BMT) involving double QDs has generated significant attention^58–61^. The BMT is particularly interesting for it has a simple structure, and also for it enhances the transport and thermo-electrical properties in both the Kondo regime^62,63^ and Coulomb blockade regime^64^. Furthermore, extensive investigations have also been carried on the some other phenomena such as the Quantum interference effect^65^ and negative tunneling Magnetoresistance^66^. The negative tunneling conductivity has been studied both experimentally^67–69^ and also theoretically^70,71^. The recent work by Masolva et al.^72^ has demonstrated that the interplay between Coulomb correlations and the asymmetry in tunneling rates gives rise to negative tunneling conductivity.

A molecular device can also be used as a spin filtering device which generates a spin-polarized current. To accomplish this, various configurations have been investigated, one of the commonly employed experimental methods being direct injection of spin-polarized charge carriers^73,74^. Various magnetic materials, including ferromagnetic metals^75–77^ and diluted magnetic semiconductors^78,79^ have been utilized as sources and drains for spin injection. Also, hybrid systems combining ferromagnets and semiconductors have been explored as alternative strategies^80–84^. But the presence of magnetic leads may cause undesirable effect because of its own magnetic field, so the use of non-magnetic leads has started gaining more focus^85–89^.

In the present work, we are interested in quantum magneto-transport in a dissipative bi-molecular transistor (BMT) which may also be referred to as a QD-dimer (QDD) transistor. We calculate, in particular, the Tunneling Current, Spectral density, differential conductance and spin polarization in the presence of e–e and e–p interactions.

## The model

**Figure 1:**
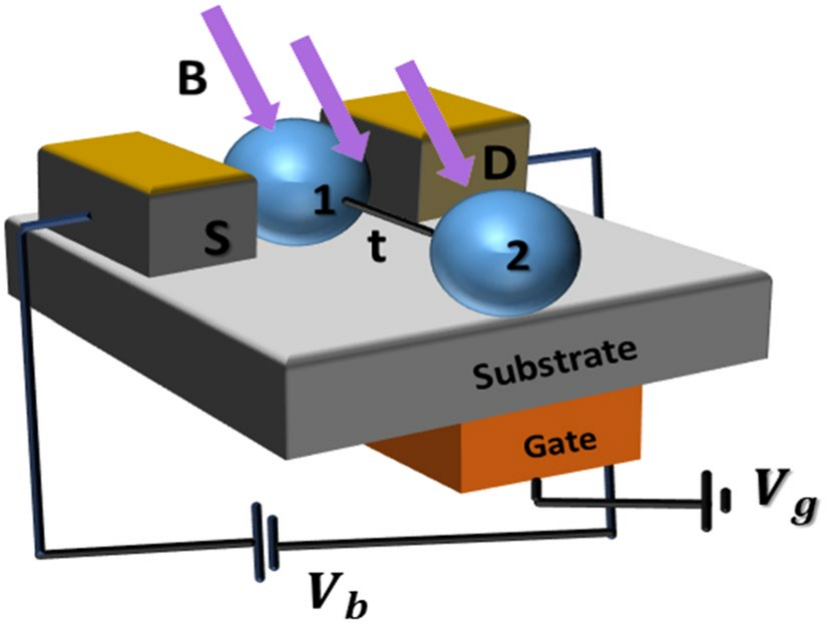
Bi-molecular transistor (BMT) using quantum dot dimer (QDD).

In Fig. 1, we present the schematic diagram of the BMT device under consideration. The central region of this device contains two QDs. The QD 1 is connected to the source (S) and the drain (D) by two metallic leads and is also connected to QD 2. Both the QDs are placed on an insulating substrate which contains a collection of uncoupled simple harmonic oscillators and acts as a phonon bath. Each quantum dot has single lattice mode that interacts with the corresponding QD electrons through e–p coupling of Holstein type. The phonons of the QDs interact with the substrate phonons though a linear coupling. We model this coupling by the Caldeira–Leggett (CL).

Hamiltonian. This interaction gives rise to a dissipative effect to the tunneling current that flows through the transistor. A magnetic field is known to have visible effect on the transport properties of a molecular transistor^90,91^. The lifting of spin degeneracy due to an external magnetic field makes a molecular transistor suitable for spin filter. The BMT system shown in Fig. 1 can be described by Hamiltonian

1 $$H = H_{l} + H_{QDD} + H_{t} + H_{B}$$

The first term in (1) i.e., $H_{l}$ represents the kinetic energy of the lead electrons i.e., the free electrons of the source (*l* = *S*) and the drain $\left( {l = D} \right)$ and is given by: $H_{l} = \mathop \sum \nolimits_{{{\varvec{k}},\sigma \in S,D}} \varepsilon_{{{\varvec{k}}\sigma }} n_{{{\varvec{k}}\sigma }} ,$ where $n_{{{\varvec{k}}\sigma }} \left( { = c_{k\sigma }^{\dag } c_{k\sigma } } \right)$ is the number operator for the S and D electrons with wave vector ${\varvec{k}}$ and spin $\sigma$ and energy $\varepsilon_{{{\varvec{k}}\sigma }}$, $c_{k\sigma }^{\dag } \left( {c_{k\sigma } } \right)$ being the corresponding creation (annihilation) operator. The second term in (1) i.e., $H_{QDD}$ describes the Hamiltonian of QDD and is given by: $H_{QDD} = H_{QDD}^{0} + H_{vib}^{0} + H_{e, vib}^{0} .$ Here, $H_{QDD}^{0}$ represents the electronic part of the QDD and is given by

2 $$H_{QDD} = \mathop \sum \limits_{i, \sigma \in QDD} \left( {\varepsilon_{i} - eV_{g} } \right)d_{i\sigma }^{\dag } d_{i\sigma } - \mathop \sum \limits_{{\left\langle {i,j} \right\rangle , \sigma \in QDD}} t_{ij} d_{i\sigma }^{\dag } d_{j\sigma } + U\mathop \sum \limits_{i \in QDD} n_{i \uparrow } n_{i \downarrow } - \frac{1}{2}\mathop \sum \limits_{i} g\mu_{B} BS_{i}^{z} ,$$

where $\varepsilon_{i}$ is the onsite energy of the electron at the $i$-th QD, $i = 1$ referring to the QD 1 and $i = 2$ referring to the QD 2, $V_{g}$ is the gate voltage, $t_{ij} = t$ is the hopping integral corresponding to hopping of an electron from one QD to the other, $n_{i\sigma } \left( { = d_{i\sigma }^{\dag } d_{i\sigma } } \right)$ is the number operator corresponding to electrons at the $i$-th QD with spin $\sigma ,$ $d_{i\sigma }^{\dag } \left( {d_{i\sigma } } \right)$ being the corresponding electron creation (annihilation) operator, $U$ is the intra-dot electron–electron (e–e) interaction strength and the last term is the total Zeeman term corresponding to the electrons of QDD, $B$ being the external magnetic field applied in the z-direction, $S_{z} ( = \frac{\hbar }{2}\mathop \sum \nolimits_{\upsigma }\upsigma {\text{d}}_{\upsigma }^{\dag } {\text{d}}_{\upsigma } )$ the total spin magnetic moment of the QD electrons, $\mu_{B}$ the Bohr magneton and $g$ the gyromagnetic ratio. $H_{vib}^{0}$ represents the vibrational degrees of freedom corresponding to two QDs of QDD and is given by: $H_{vib}^{0} = \mathop \sum \nolimits_{i \in QDD} \left[ {\frac{{p_{i}^{2} }}{{2m_{0} }} + \frac{1}{2}m_{0} \omega_{0}^{2} x_{i}^{2} } \right] ,$ where again $i = 1$ refers to QD 1 and $i = 2$ refers to QD 2, with $x_{i}$ and $p_{i}$ being respectively the coordinate and momentum of the vibrational mode of the i-th QD and $\omega_{0}$ is the dimensionless phonon frequency. $H_{e, vib}^{0}$ describes the electron–phonon (e–p) interaction for QDD and is given by: $H_{e,vib}^{0} = \mathop \sum \nolimits_{i \in QDD} g_{i} n_{i\sigma } x_{i} ,$ where $g_{i}$ is the e–p coupling constant for the $i$-th QD and since the two QDs are identical, we choose $g_{1} = g_{2} .$ The third term of Eq. (1) i.e., $H_{t}$ is the lead-QD hybridization term that is responsible for tunneling and is given by: $H_{t} = V_{r} \mathop \sum \nolimits_{\sigma } \left( {c_{S\sigma }^{\dag } d_{1\sigma } + d_{1\sigma }^{\dag } c_{S\sigma } } \right) + V_{r} \mathop \sum \nolimits_{\sigma } \left( {c_{D\sigma }^{\dag } d_{1\sigma } + d_{1\sigma }^{\dag } c_{D\sigma } } \right),$ where $V_{r}$ denotes the QD-lead hybridization strength. Finally, $H_{B}$ in (1) describes the substrate oscillators and the interaction between the substrate oscillators and the QD phonons which is modeled by the linear Caldeira–Leggett model:

3 $$H_{B} = H_{B}^{0} + H_{vib - B}^{0} = \mathop \sum \limits_{j = 1,2, .. N \in B} \left[ {\frac{{P_{j}^{2} }}{{2m_{0} }} + \frac{1}{2}m_{0} \omega_{j}^{2} \chi_{j}^{2} } \right] + \mathop \sum \limits_{{\begin{array}{*{20}c} {j = 1,2, N \in B} \\ {i = 1,2 \in QDD} \\ \end{array} }} \beta_{j} x_{i} \chi_{j} ,$$

where $\left( {\chi_{j} , P_{j} } \right)$ are the canonical conjugate variables corresponding to bath oscillators, $\omega_{j}$ is the frequency of the $j$-th bath oscillator and $\beta_{j}$ is the coupling constant between the QDD oscillators and the $j$-th bath oscillator.

## Formulation

### Elimination of QDD-bath interaction

To eliminate the QDD-bath interaction, we apply a transformation: $\tilde{\chi }_{j} = \left[ {\chi_{j} + \beta_{j} \left( {\mathop \sum \nolimits_{i = 1,2} x_{i} } \right)/m_{j} \omega_{j}^{2} } \right] ; \tilde{P}_{j} = - i\hbar \left( {\partial /\partial \tilde{\chi }_{j} } \right).$ The Hamiltonian $\left( {H_{vib}^{0} + H_{B} } \right)$ can then be approximately written as

4 $$H_{vib}^{0} + H_{B} = \mathop \sum \limits_{i , \sigma \in QDD} \left[ {\frac{{p_{i}^{2} }}{{2m_{0} }} + \frac{1}{2}m_{0} \tilde{\omega }_{0}^{2} x_{i}^{2} } \right] + \mathop \sum \limits_{j = 1,2, \ldots N} \left[ {\frac{{\tilde{P}_{j}^{2} }}{{2m_{j} }} + \frac{1}{2}m_{j} \omega_{j}^{2} \tilde{\chi }_{j}^{2} } \right] ,$$

where $\tilde{\omega }_{0} = \left( {\omega_{0}^{2} - \Delta \omega^{2} } \right)^{1/2} ; \;\Delta \omega^{2} = \mathop \sum \nolimits_{j} \left( {\beta_{j}^{2} /m_{0} m_{j} \omega_{j}^{2} } \right)$ and we have assumed that the substrate-induced correlation of the QDD phonons which is of the order of $\beta_{j}^{2}$ can be neglected. Assuming that the bath oscillators are fully characterized by a spectral function $J\left( \omega \right),$ we can convert, in the large-N limit, the summation over $j$ in $\Delta \omega^{2}$ into an integral over $\omega$ through the density of states $J\left( \omega \right).$ $\tilde{\omega }_{0}^{2}$ can then be written as :$\tilde{\omega }_{0}^{2} = \omega_{0}^{2} - \frac{2}{{m_{0} }}\smallint \left( {J\left( \omega \right)/\omega } \right) d\omega .$ After comparison: $J\left( \omega \right) = \mathop \sum \nolimits_{j}^{N} \left( {\beta_{j}^{2} /2m_{j} \omega_{j}^{2} } \right)\delta \left( {\omega - \omega_{j} } \right) ,$ which in the large-N limit can be written in the Lorentz–Drude form as: $J\left( \omega \right) = 2m_{0} \gamma \omega \left[ {1 + \left( {\omega /\omega_{c} } \right)^{2} } \right],$ where $\gamma$ is the damping rate and $\omega_{c}$ is the cutoff frequency. We assume that the frequency of the system is much smaller than $\omega_{c}$ so that we can write: $\tilde{\omega }_{0}^{2} = \omega_{0}^{2} - \smallint d\omega J\left( \omega \right)\left[ {2J\left( \omega \right)/m_{0} \omega } \right] = \left( {\omega_{0}^{2} - 2\pi \gamma \omega_{c} } \right).$ Thus the Hamiltonian for the BMT devices reduces to

5 $$\begin{aligned} \overline{H} & = \mathop \sum \limits_{{{\varvec{k}},\sigma \in S,D}} \varepsilon_{{{\varvec{k}}\sigma }} n_{{{\varvec{k}}\sigma }} + \mathop \sum \limits_{L = S,D,\sigma } \left( {V_{r} c_{L\sigma }^{\dag } d_{1\sigma } + V_{r}^{\dag } d_{1\sigma }^{\dag } c_{L\sigma } } \right) + \mathop \sum \limits_{i, \sigma \in QD} \left( {\epsilon_{i} - eV_{g} } \right)d_{i\sigma }^{\dag } d_{i\sigma } - \mathop \sum \limits_{{\left\langle {i,j} \right\rangle , \sigma \in QD}} t d_{i\sigma }^{\dag } d_{j\sigma } \\ & \quad + U\mathop \sum \limits_{i \in QD} n_{i \uparrow } n_{i \downarrow } + \hbar \tilde{\omega }_{0} \mathop \sum \limits_{i \in QD} b_{i}^{\dag } b_{i} + \mathop \sum \limits_{i \in QD} \tilde{g}_{{\varvec{i}}} d_{i\sigma }^{\dag } d_{i\sigma } \left( {b_{i} + b_{i}^{\dag } } \right) + \frac{1}{2}\mathop \sum \limits_{i = 1,2} g\mu_{B} BS_{i,z} , \\ \end{aligned}$$

where $b_{i}^{\dag } \;{\text{and}}\;b_{i}$ are creation and annihilation operators for the renormalized phonons of QDD, $\tilde{g}_{i}$ being the renormalized e–p coupling constant. We have assumed here that the primary role of the heat bath is to reduce the phonon frequency leading to a resistive effect akin to dissipation. All higher order effects have been neglected.

### Decoupling of e–p interaction

Our next step is to deal with the e–p interaction. One of the most important effects of the e–p interaction is to transform the phonon vacuum into a coherent phonon state. This can be accomplished by employing the celebrated Lang–Firsov transformation^45^ $e^{S}$ with: $S = \mathop \sum \nolimits_{i\sigma } \lambda_{i} n_{i\sigma } \left( {b_{i}^{\dag } - b_{i} } \right) ,\;\lambda_{i} = \left( {\tilde{g}_{i} /\hbar \omega_{0} } \right) ,\;\lambda_{1} = \lambda_{2} = \lambda .$ The transformed Hamiltonian is given by

6 $$\begin{aligned} \tilde{H} & = \mathop \sum \limits_{{{\varvec{k}},\sigma \in S,D}} \varepsilon_{{{\varvec{k}}\sigma }} n_{{{\varvec{k}}\sigma }} + \mathop \sum \limits_{L = S,D,\sigma } \left( {\tilde{V}c_{L\sigma }^{\dag } d_{1\sigma } + \tilde{V}c_{D\sigma }^{\dag } d_{1\sigma } } \right) + \mathop \sum \limits_{i, \sigma \in QDD} \tilde{\varepsilon }_{i} d_{i\sigma }^{\dag } d_{i\sigma } - \mathop \sum \limits_{{\left\langle {i,j} \right\rangle }} \tilde{t}d_{i\sigma }^{\dag } d_{j\sigma } + \tilde{U}\mathop \sum \limits_{i \in QDD} n_{i \uparrow } n_{i \downarrow } \\ & \quad + \hbar \tilde{\omega }_{0} \mathop \sum \limits_{i} b_{i}^{\dag } b_{i} + \frac{1}{2}g\mu_{B} BS_{d}^{z} \\ \end{aligned}$$

where $\tilde{\varepsilon }_{i} , \tilde{U}$ and $\tilde{t}$ are e–p interaction-induced renormalized parameters and are given by:$\tilde{\varepsilon }_{i} = \varepsilon_{i} - eV_{g} - \hbar \tilde{\omega }_{0} \lambda^{2} ,\;\tilde{U} = \left( {U - \hbar \tilde{\omega }_{0} \lambda^{2} } \right),\;\tilde{V} = V_{r} X_{i} ,\;\tilde{t} = tX_{i}^{\dag } X_{j} ,\;X_{i} = e^{{ - \lambda \left( {b_{i}^{\dag } - b_{i} } \right)}} .$

### Tunneling current, differential conductance and spin polarization

In order to calculate the tunneling current we first calculate the current from source to QD ($$J_{L} )$$ which is given by the time derivative of the occupation number operator for the source electrons, $$N_{s} = \mathop \sum \nolimits_{k,\sigma } \epsilon_{k} c_{Sk\sigma }^{\dag } c_{Sk\sigma }$$. Thus we have: $$J_{S} = - \left( {ie/\hbar } \right)0\left\langle {{|}\left[ {N_{s} ,\tilde{H}} \right]{|}0} \right\rangle = \left( {2e/\hbar } \right)\mathop \sum \nolimits_{{k_{x} \sigma }} VRe\left( { G_{{1\sigma ,k_{x} \sigma }}^{ < } } \right),$$ where the averaging state $$\left| 0 \right\rangle$$ is the ground state (GS) of the whole system i.e., $$\left| 0 \right\rangle = \left| 0 \right\rangle_{el} \left| 0 \right\rangle_{ph}$$, $$|0_{el}$$ being the GS of the electron sub-system and $$\left| 0 \right\rangle_{ph}$$ that of the phonon sub-system and $$G_{{1\sigma ,k_{x} \sigma }}^{ < }$$ is the Keldysh lesser (tunneling) Green function which is given by: $$G_{{{\varvec{k}}\sigma ,1\sigma }}^{ < } \left( {t,t^{\prime}} \right) = i\left\langle {0|d_{1o}^{\dag } \left( {t^{\prime}} \right)c_{{{\varvec{k}}\sigma }} \left( t \right)|0} \right\rangle .$$ The Keldysh greater (tunneling) Green function is defined as: $$G_{{1\sigma ,{\varvec{k}}\sigma }}^{ > } \left( {t,t^{\prime}} \right) = i\left\langle {0|d_{1\sigma } \left( t \right)c_{{{\varvec{k}}\sigma }}^{\dag } \left( {t^{\prime}} \right)|0)} \right\rangle$$ with the property: $$G_{{\user2{k\sigma },d}}^{ < } \left( {t,t} \right) = - \left[ {G_{{{\varvec{d}},\user2{k\sigma }}}^{ < } \left( {t,t} \right)} \right]^{*} .$$ Using the equation of motion technique and Langreth’s analytical continuation, the source current can be calculated as

7 $$J_{S\sigma } = \frac{ie}{{2\pi h}}\smallint {\Gamma }_{S} \left( {\epsilon } \right)\left[ {G_{1\sigma ,1\sigma }^{ < } \left( \varepsilon \right) + f_{S} \left( {\epsilon } \right)\left( {G_{1\sigma ,1\sigma }^{r} \left( \varepsilon \right) - G_{1\sigma ,1\sigma }^{a} \left( \varepsilon \right)} \right)} \right]d\varepsilon .$$

The bias voltage ($eV_{b}$) and the mid-voltage ($eV_{m}$) are related to the chemical potential by the relation: $\mu_{L} - \mu_{R} = eV_{b} , \left( {\mu_{L} + \mu_{R} } \right)/2 = eV_{m}$ and $\Gamma_{S} \left( {\epsilon } \right)$ measures the hybridization of the QD 1 with the source (drain) and is given by $\Gamma_{S} \left( \varepsilon \right) = 2\pi \varrho_{S} \left( \varepsilon \right)\overline{{\tilde{V}}}_{k} V_{k}^{*} ,$ $\rho_{S\left( D \right)}$ being the density of energy states in S(D) and $G_{11}^{r\left( a \right)} \left( {\epsilon } \right)$ is the retarded (advanced) QD Green function in the energy space and can be obtained from the corresponding time-dependent Green function $G_{11}^{r\left( a \right)} \left( {\tau = t - t^{\prime}} \right)$ by Fourier transformation. $G_{dd}^{r\left( a \right)} \left( \tau \right)$ is defined as :$G_{1\sigma ,1\sigma }^{r\left( a \right)} \left( \tau \right) = \mp i \theta \left( { \pm t \mp t^{\prime}} \right)\left\langle {0\left| {\left\{ {\tilde{d}_{1} \left( t \right),\tilde{d}_{1}^{\dag } \left( {t^{\prime}} \right)} \right\}} \right|0} \right\rangle$. In a similar way, the drain current is obtained as

8 $$J_{D\sigma } = \frac{ie}{{2\pi h}}\smallint \Gamma_{D} \left( {\epsilon } \right)\left[ {G_{1\sigma ,1\sigma }^{ < } \left( \varepsilon \right) + f_{D} \left( {\epsilon } \right)\left( {G_{1\sigma ,1\sigma }^{r} \left( \varepsilon \right) - G_{1\sigma ,1\sigma }^{a} \left( \varepsilon \right)} \right)} \right]d\epsilon .$$

In the steady state, the current will be uniform, so that ${ }J = J_{S} = - J_{D}$, and after symmetrizing, the total current can be written as

9 $$J = \frac{{J_{S} - J_{D} }}{2} = \frac{e}{h}\left[ {\smallint d\epsilon \left[ {f_{S} \left( {\epsilon } \right)\Gamma_{S} \left( {\epsilon } \right) - f_{D} \left( {\epsilon } \right)\Gamma_{D} \left( {\epsilon } \right)} \right]A\left( {\epsilon } \right)} \right] + \smallint d\epsilon \left[ {\Gamma_{S} \left( {\epsilon } \right) - \Gamma_{D} \left( {\epsilon } \right)} \right]G^{r}_{1\sigma ,1\sigma } \left( {\epsilon } \right).$$

The tunneling current for the symmetric coupling $\left( {\Gamma_{S} \left( {\epsilon } \right) = \Gamma_{D} \left( {\epsilon } \right) = \Gamma } \right)$ and in wide band limit (when $\Gamma \left( {\epsilon } \right)$ is independent of energy) can be written as^89^

10 $$J = \frac{e\Gamma }{h}\smallint d\epsilon \left[ {f_{S} \left( {\epsilon } \right) - f_{D} \left( {\epsilon } \right)} \right]A\left( {\epsilon } \right),$$

where $f_{S,D} \left( {\epsilon } \right)$ denotes the Fermi distribution function for S(D) electrons: $f_{S\left( D \right)} \left( {\epsilon } \right) = \left[ {e^{{\left( {\epsilon - \mu_{S\left( D \right)} } \right)/k_{B} T_{S\left( D \right)} }} + 1} \right]^{ - 1} ,$ $\mu_{S\left( D \right)}$ and $k_{B} T_{S\left( D \right)}$ being respectively the chemical potential and the thermal energy of the source (drain) and ${\text{A}}\left( \varepsilon \right)$ is the spectral function which describes the excitations and is related to the Green functions as: $A\left( {\epsilon } \right) = i\left[ {G_{11}^{r} \left( {\epsilon } \right) - G_{11}^{a} \left( {\epsilon } \right)} \right] = i\left[ {G_{11}^{ < } \left( {\epsilon } \right) - G_{11}^{ > } \left( {\epsilon } \right)} \right],$ where $G_{11}^{r\left( a \right)} \left( {\epsilon } \right)$ refers to the retarded (advanced) Green function and $G_{11}^{ < \left( > \right)} \left( {\epsilon } \right)$ the lesser (greater) Green function for the QD 1 in the energy space and can be obtained by Fourier transformation from the corresponding time-dependent Green functions $G_{11}^{r\left( a \right)} \left( {t - t^{\prime}} \right)$ and $G_{11}^{ < \left( > \right)} \left( {t - t^{\prime}} \right)$ which can be written as

11 $$\begin{aligned} G_{11}^{r\left( a \right)} \left( {t - t^{\prime}} \right) & = \mp i\theta \left( {t - t^{\prime}} \right)\left\langle {\left\{ {d_{1} \left( t \right),d_{1}^{\dag } \left( {t^{\prime}} \right)} \right\}} \right\rangle \left\langle {\left\{ {X_{1}^{\dag } \left( t \right)X_{1} \left( {t^{\prime}} \right)} \right\}} \right\rangle_{ph} \\ G_{11}^{ > } \left( {t - t^{\prime}} \right) & = - i\left\langle {d_{1} \left( t \right)d_{1}^{\dag } \left( {t^{\prime}} \right)} \right\rangle \left\langle {X_{1}^{\dag } \left( {t^{\prime}} \right)X_{1} \left( t \right)} \right\rangle_{ph} ,\;G_{11}^{ < } \left( {t - t^{\prime}} \right) = i\left\langle {d_{1}^{\dag } \left( {t^{\prime}} \right)d_{1} \left( t \right)} \right\rangle \left\langle {X_{1}^{\dag } \left( t \right)X_{1} \left( {t^{\prime}} \right)} \right\rangle_{ph} , \\ \end{aligned}$$

The phononic part is given by: $\left\langle {X_{1}^{\dag } \left( t \right)X_{1} \left( {t^{\prime}} \right)} \right\rangle_{ph} = \mathop \sum \nolimits_{l = - \infty }^{\infty } L_{l} e^{{il\tilde{\omega }_{0} \left( {t - t^{\prime}} \right)}}$^92^, where $L_{l} = e^{{ - \lambda^{2} \left( {1 + 2N_{ph} } \right)}} \times$.

$\mathop \sum \nolimits_{l = - \infty }^{\infty } I_{l} \left( {z^{\prime}} \right),\exp \left( {l\beta \hbar \tilde{\omega }_{0} /2} \right),$ and $I_{l} \left( {z^{\prime}} \right)$ is the *l-* th order Bessel function with ${ }z^{\prime} = 2\lambda^{2} \sqrt {N_{ph} \left( {N_{ph} + 1} \right)} .$ Thus, the lesser and greater Greens functions can be expanded as: $G_{11}^{ < ( > )} \left( {t - t^{\prime}} \right) = \tilde{G}_{11}^{ < ( > )} \left( {t - t^{\prime}} \right)\mathop \sum \nolimits_{l = - \infty }^{\infty } L_{l} e^{{il\tilde{\omega }_{0} \left( {t - t^{\prime}} \right)}} ,$ and $G_{11}^{ < \left( > \right)} \left( {\epsilon } \right)$ is given by: $G_{11}^{ < ( > )} \left( {\epsilon } \right) = \mathop \sum \nolimits_{l = - \infty }^{\infty } L_{l} \tilde{G}_{11}^{ < ( > )} \left( {\epsilon \mp l\hbar \tilde{\omega }_{0} } \right).$ At $T = 0K$, $L_{l} = e^{{ - \lambda^{2} }} \lambda^{l} /l!$ for n ≥ 0 and $L_{l} = 0$ for n < 0. $A\left( {\epsilon } \right)$ is thus given by:

12 $$A\left( {\epsilon } \right) = \mathop \sum \limits_{l = - \infty }^{\infty } iL_{l} \left[ { \tilde{G}_{11}^{ > } \left( {\epsilon - l\hbar \tilde{\omega }_{0} } \right) - \tilde{G}_{11}^{ < } \left( {\epsilon + l\hbar \tilde{\omega }_{0} } \right)} \right] = \mathop \sum \limits_{l = - \infty }^{\infty } iL_{l} \left[ { \tilde{G}_{11}^{ > } \left( \varepsilon \right) - \tilde{G}_{11}^{ < } \left( \varepsilon \right)} \right],$$

where* l* gives the number of phonons and $\varepsilon = \epsilon - l\hbar \tilde{\omega }_{0}$. To calculate $\tilde{G}_{11}^{ < } \left( \varepsilon \right) \;{\text{and}}\;\tilde{G}_{11}^{ > } \left( \varepsilon \right),$ we will use Keldysh formalism where $\tilde{G}_{11}^{ < ( > )} \left( \varepsilon \right)$ is given by: $\tilde{G} _{11}^{ < ( > )} \left( \varepsilon \right) = \tilde{G}_{11}^{r} \left( \varepsilon \right){\Sigma }^{ < \left( > \right)} \tilde{G}_{11}^{a} \left( \varepsilon \right),$ where ${\Sigma }^{ < } \left( \varepsilon \right) = \mathop \sum \nolimits_{{\alpha \in \left( {S,D} \right)}} V_{\alpha }^{*} g_{\alpha }^{ < } \left( {\epsilon } \right)V_{\alpha }$ is the self-energy, describing the coupling between source and quantum dot and quantum dot and drain and is calculated using the Green function: $\tilde{G}_{\alpha }^{ < } \left( {t - t^{\prime}} \right) = i\left\langle {c_{\alpha }^{\dag } \left( t \right)c_{\alpha } \left( {t^{\prime}} \right)} \right\rangle = if\left( {\epsilon } \right)\exp \left( { - i\epsilon_{\alpha } \left( {t - t^{\prime}} \right)} \right)$ where $\tilde{G}_{\upalpha }^{ < } \left( {t - {\text{t}}^{\prime } } \right)$ is the lesser Green function of source and drain. Therefore self-energy is given by:${\Sigma }^{ < } \left( \varepsilon \right) = \mathop \sum \nolimits_{k\alpha } \left( {V_{\alpha } } \right)^{2} g_{\alpha }^{ < } \left( \varepsilon \right) = i\Gamma \left[ { f_{S} \left( \varepsilon \right) + f_{D} \left( \varepsilon \right).} \right]$ Similarly ${\Sigma }^{ > } \left( \varepsilon \right) = i\Gamma \left[ {2 - f_{s} \left( \varepsilon \right) - f_{D} \left( \varepsilon \right)} \right].$ After calculating the self-energy, we calculate the retarded and advanced Green function $\tilde{G}_{11}^{r\left( a \right)}$ using the equation of motion technique:

13 $$\begin{aligned} i\frac{\partial }{{\partial t^{\prime}}}\left[ { \tilde{G}_{11}^{{{\text{r}}\left( {\text{a}} \right)}} \left( \varepsilon \right)} \right]_{el} & = \delta \left( { \pm t \mp t^{\prime}} \right) \mp i\theta \left( { \pm t \mp t^{\prime}} \right)\left\{ {d_{1} \left( t \right),\left[ {d_{1}^{\dag } ,\tilde{H} } \right]} \right\} \\ & = \delta \left( { \pm t \mp t^{\prime}} \right) \\ \end{aligned}$$

14 $$\begin{aligned} & \mp i\theta \left( { \pm t \mp t^{\prime}} \right)\left\langle {\left\{ {d_{{1\sigma^{\prime}}} \left( t \right),\left[ {d_{{1\sigma^{\prime}}}^{\dag } \left( {t^{\prime}} \right) , \mathop \sum \limits_{{{\varvec{k}},\sigma \in S,D}} \varepsilon_{{{\varvec{k}}\sigma }} n_{{{\varvec{k}}\sigma }} + \mathop \sum \limits_{i , \sigma \in QDD} \left( {\epsilon_{0} + \tilde{U}\left\langle {n_{i} } \right\rangle } \right)n_{i\sigma } } \right.} \right.} \right. \\ & \quad \left. {\left. {\left. { + \mathop \sum \limits_{\sigma } \left( {\tilde{V}c_{S\sigma }^{\dag } d_{1\sigma } + \tilde{V}^{\dag } d_{1\sigma }^{\dag } c_{S\sigma } } \right) + \mathop \sum \limits_{\sigma } \left( {\tilde{V}c_{D\sigma }^{\dag } d_{1\sigma } + d_{1\sigma }^{\dag } \tilde{V}^{\dag } c_{D\sigma } } \right) - \mathop \sum \limits_{\sigma } \tilde{t}\left( {d_{1\sigma }^{\dag } d_{2\sigma } + d_{2\sigma }^{\dag } d_{1\sigma } } \right)} \right]} \right\}} \right\rangle \\ \end{aligned}$$

which on simplification gives

15 $$\tilde{G}_{11 \downarrow \uparrow }^{r\left( a \right)} \left( \varepsilon \right) = \frac{{\epsilon - \tilde{\varepsilon } \pm \mu_{B} B - \tilde{U}\left\langle n \right\rangle - V_{g} }}{{\left( {\epsilon \pm \mu_{B} B - \tilde{\varepsilon } - \tilde{U}\left\langle n \right\rangle - V_{g} } \right)\left( {\left( {\epsilon - \tilde{\varepsilon } \pm \mu_{B} B - \tilde{U}\left\langle n \right\rangle - V_{g} \pm i{\Gamma }} \right)} \right) - \left( {te^{{\lambda^{2} }} } \right)^{2} }} ,$$

We calculate the spectral function using relation (12) and (15) and then the tunneling current from Eq. (10). Finally we calculate the Differential Conductance (= $dJ/dV$) and the spin polarization parameter: $P_{\sigma , - \sigma } \left( { = \left( {J_{\sigma } - J_{ - \sigma } } \right)/\left( {J_{\sigma } + J_{ - \sigma } } \right)} \right)$.

## Result and discussion

**Figure 2:**
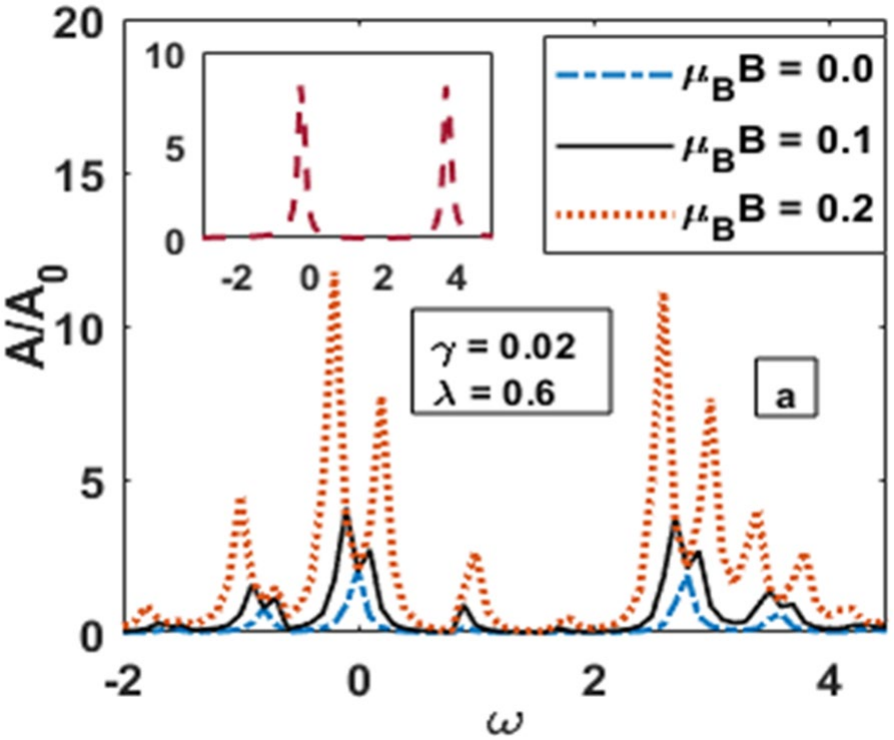
Spectral Function versus ω for different value of *μ*_*B*_*B*.

We have assumed that the QD1 is symmetrically connected with the source and the drain. We consider the energy level of each QD as zero and measure the energy in units of phonon energy $\hbar \omega_{0 }$. For most part of our calculation we consider $\Gamma = 2,\; eV_{g} = - 1.5, \;1 eV_{m} = 0.1, e\;V_{b } = 0.5 \;{\text{and}}\; U = 5$. We deal with the Coulomb interaction using the mean-field Hartree–Fock approximation. Because of the e–p interaction, the onsite Coulomb interaction energy *U* is substantially reduced due to the polaronic effect and the use of mean-field approximation is justified for not too large Coulomb interaction. Thus our results will be well outside the Kondo regime. We also assume that the electron charge density in both the source and drain is constant. Figure 2 displays the behaviour of the spectral function (SF) of QDD, in the presence of e–e interaction, e–p interaction, magnetic field and dissipation. The inset shows the behaviour of SF in the absence of e–p interaction, damping and the magnetic field. One can clearly see the presence of two Lorentzian peaks. For non-zero e–p interaction and damping due to the substrate, side bands appear along with the Lorentzian peaks due to polaronic effect. As the magnetic field is introduced, these peaks split and increase with the increase in magnetic field.

**Figure 3:**
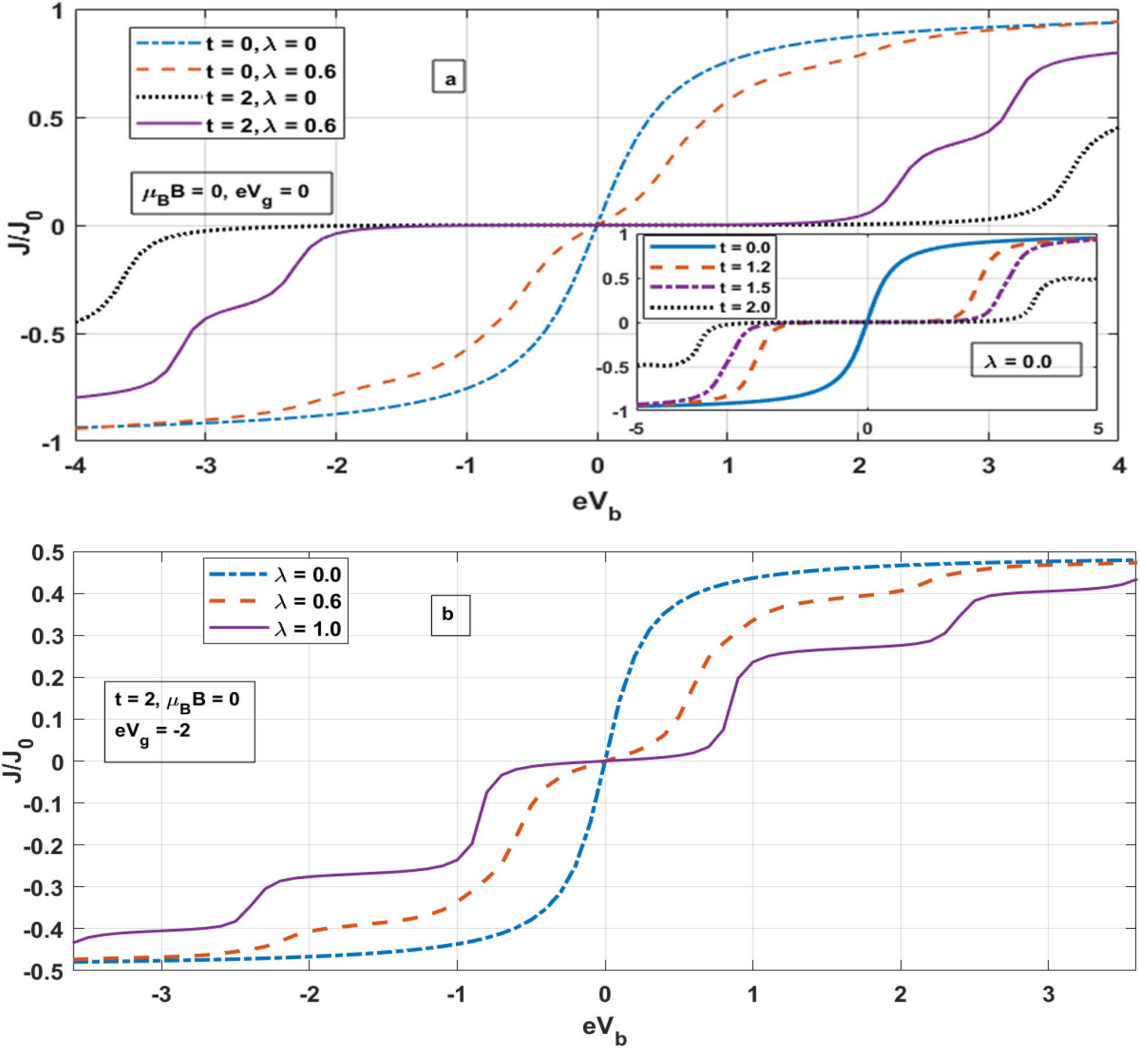
*J* versus eV_b_ for different system parameters.

In Fig. 3a, we show the behaviour of tunneling current as a function of the bias voltage for $B = 0\; {\text{and}}\;eV_{g} = 0,$. We consider four cases. In Case 1, we take $t = 0$ and $\lambda = 0$ i.e. when there is only one QD present in the system and there is no e–p interaction. In that case we observe that as the bias voltage increases, tunneling current increases and have an ohmic nature at low bias voltage. As the bias voltage increases further, the tunneling current shows a non-ohmic behaviour and eventually saturates. This happens because of the following reason. As we increase the bias voltage, the Fermi level of the source rises, which increases the probability of tunneling of electrons from the source to the QD leading to the increase in the current. However, the QD can accommodate only a limited number of electrons and so above a certain voltage, the current saturates. In Case 2, $t = 2,$ ${\uplambda } = 0 ,$ i.e., when we have both the QDs contributing to the system in the absence of e–p interaction, the tunneling current becomes much smaller and develops a stair-like structure. The step-like behavior observed in the electronic transport can be attributed to various contributions that arise from transitions of electrons with different energies in presence of Coulomb correlations^84^. At low voltage, on one hand, the electron flow from the source to the QD1 is low and on the other hand, because of large *t*, even majority of those electrons flow to the second QD giving rise to zero current. This happens till the second QD levels get saturated. As the bias voltage is further increased, electrons tunnel from QD1 to the drain leading to a sudden hike in the current and this increase in current continues till QD1 is saturated. Above this voltage, the current exhibits a plateau. The behaviour of the current as a function of the bias voltage for different values of *t* in the absence of e–p interaction is shown in the inset of Fig. 3a. In the third case i.e., when $t = 2$, and $\lambda = 0.6$, we find that the current is higher compared to the case 2. This is because both *t* and *λ* reduce the current, but *t* has a stronger effect. So when *λ* is non-zero, the effective *t* may be very small because of the Holstein reduction factor arising due to polaron formation. Therefore the current in the case of $t = 2$, and $\lambda = 0.6$ is larger than in the case of $t = 2,$ ${\uplambda } = 0.$ However, this figure needs a more deeper interpretation. Because of nonzero *λ*, both the hybridization coefficient $V_{r}$ and the hopping parameter *t* are reduced and so the probability of electrons tunneling from the source to QD1 and from QD1 to drain and also the hopping of electrons from QD1 to QD2 also decreases. As a result, current becomes zero at low bias voltage. As the bias voltage exceeds a certain value, it can dominate over the reduction effect of e–p interaction and then current starts increasing. During this period electrons flow from Q1 to both QD2 and the drain. The process continues till QD2 is saturated. At this stage, because of Coulomb blockade, current remains same till the bias voltage does not become large enough to allow electron to tunnel from the source to QD1. This gives the second plateau at a finite current. As the bias voltage is increased above this value, current starts increasing and now electrons flow only to the drain as the QD2 is already saturated. The current keeps increasing with the bias voltage until the QD2 is saturated. Above this voltage, current saturates again and we get the third plateau. The three-stair structure occurs due to the combination of the e–p interaction effect and the Coulomb blockade. In Case 4, we can see the same qualitative behaviour but the effect is feeble because of zero value of the hopping coefficient. In Fig. 3b we plot the current as a function of the bias voltage for different values of e–p interaction at a fixed gate voltage. The dependence of the tunneling current on gate voltage is shown in inset. The main figure shows that as the e–p interaction increases, the current decreases. The stair-structure can be explained using the same logic as used for the Fig. 3a. Also one can observe a negative differential resistance (NDR) in a particular region between 4.5 and 5 eVb in the insert figure. Several explanations have been suggested for NDR. These include renormalization of molecular energy levels due to applied bias, electron-phonnon and electron–electron interactions, and current induced forces.

**Figure 4:**
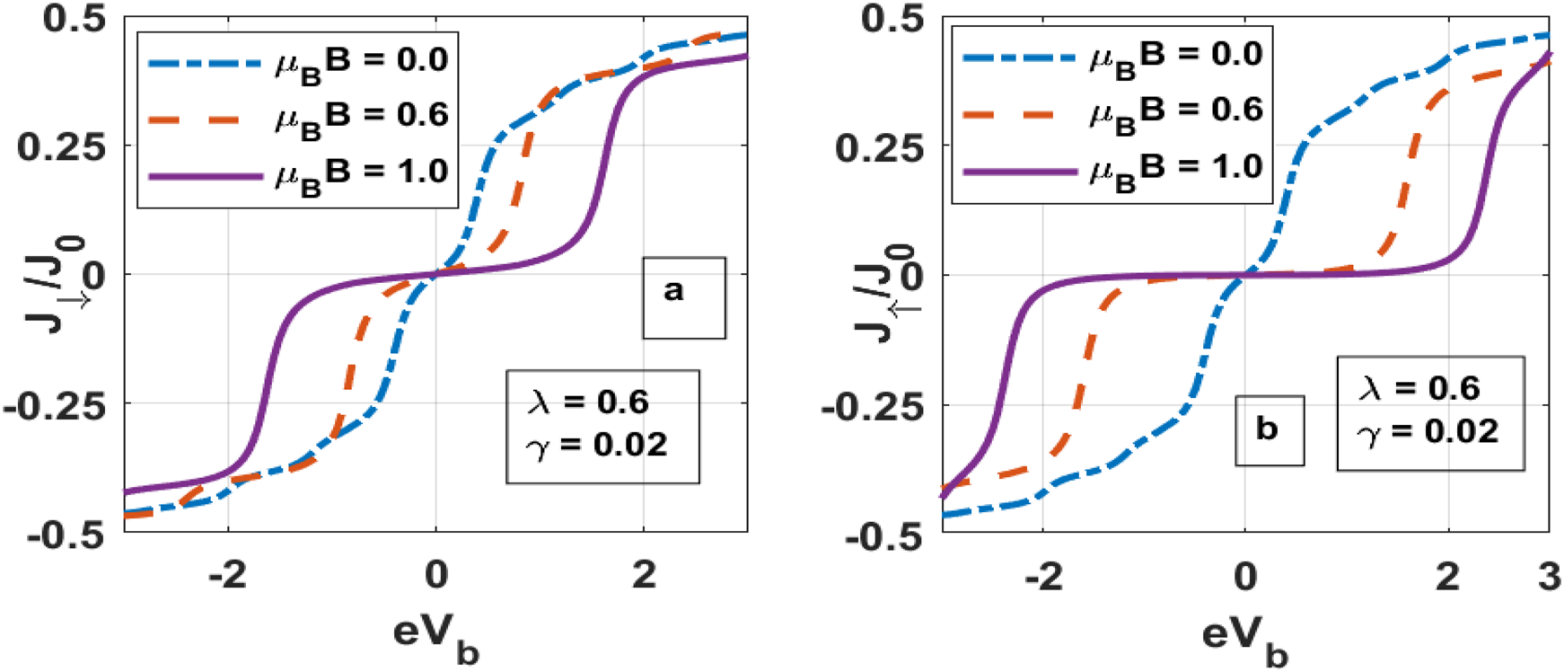
*J*_↓_ and *J*_↑_ versus eV_b_ for different values of μ_B_B.

In Fig. 4 we have shown the spin-up and spin-down tunneling currents (${\text{J}}_{ \uparrow } \;{\text{and}}\;{\text{J}}_{ \downarrow }$) as a function of the bias voltage for different value of $\mu_{B} B$. As the bias voltage is increased, more number of electrons tunnel from the source to the quantum dot and hence the current increases. Of course, as the QD can accommodate only a certain number of electrons, beyond a certain bias voltage, the current saturates. In the presence of a magnetic field the spin-degeneracy of the QD states is lifted and the spin-down states shift up and the spin-up states shift down. As a result, the spin-down electrons find it difficult to tunnel from source to the QD and consequently the spin-down current decreases with increasing magnetic field (Fig. 4a). On the other hand, with increasing magnetic field, the up-spin levels go down and then the QD electrons find it difficult to tunnel from QD to the drain unless the bias voltage is high (Fig. 4b). Thus again, the spin-up current also decreases with the increase in the magnetic field. In the case of a high magnetic field, both the spin-up and spin-down currents remain essentially zero unless the bias voltage is higher than a critical value. This is because at a high magnetic field, the spin-down level may be much higher than the Fermi level of the source and the spin-up level much lower than the Fermi level of the drain, making both the spin-up and spin-down currents zero.

**Figure 5:**
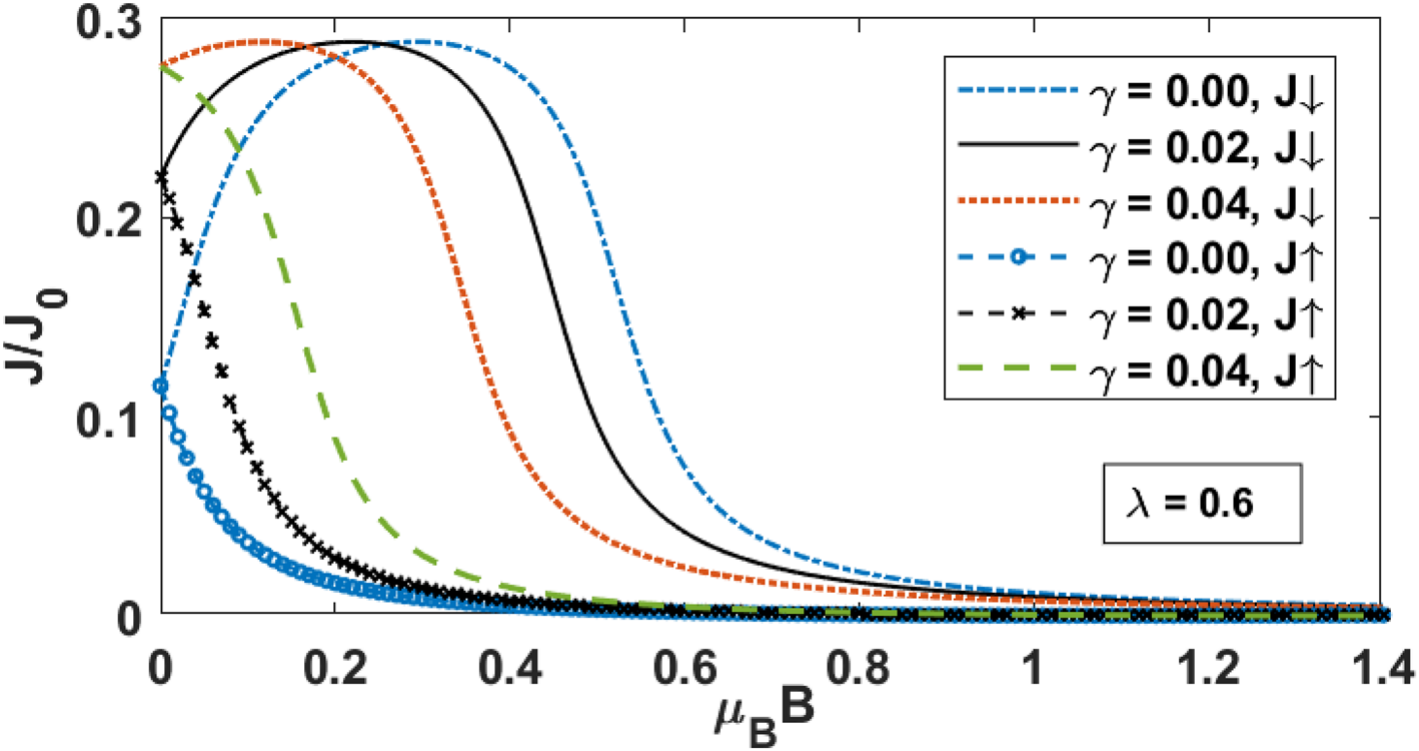
J_↑_ and J_↓_ versus *μ*_*B*_B for different values of γ.

In Fig. 5, the spin-up and spin-down tunneling currents are plotted as a function of the magnetic field with different values of the damping rate. In the absence of substrate-QD interaction, as the magnetic field is increased, ${\text{J}}_{ \downarrow }$ increases (as can also be seen from Fig. 4) initially, then attains a maxima and finally decreases to zero. The spin-up current, however, monotonically decreases with the magnetic field. These characteristics are consistent with the results shown in Fig. 4. When the substrate-QD interaction is involved, the energy levels of the QDs get renormalized, i.e., spin-up and spin-down levels move higher. As the magnetic field increases, the spin-down energy level increases. This further increment causes peaks to move to the lower value of the magnetic field. Similarly, with the increasing magnetic field, the spin-up energy level shifts down, but substrate-QD interaction moves it higher, causing a slight increase in the current.

**Figure 6:**
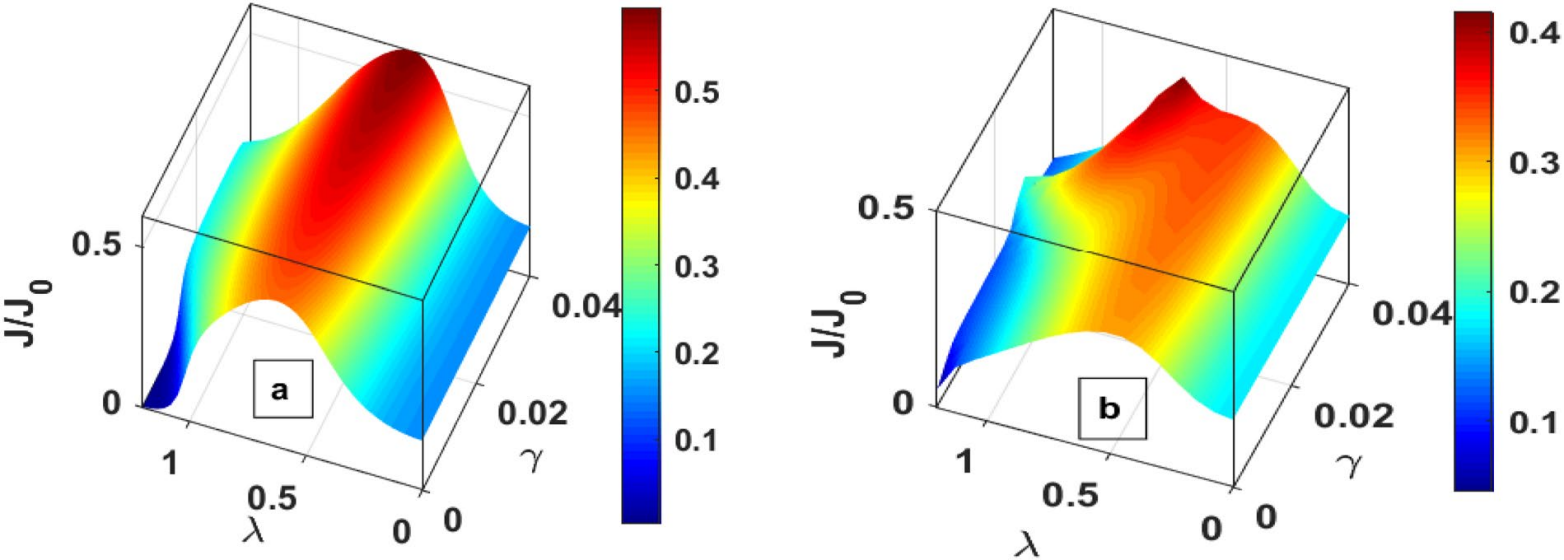
Three-dimentional plot of J as a function of λ and γ (**a**) *μ*_*B*_*B* = 0 (**b**) *μ*_*B*_*B* ≠ 0.

**Figure 7:**
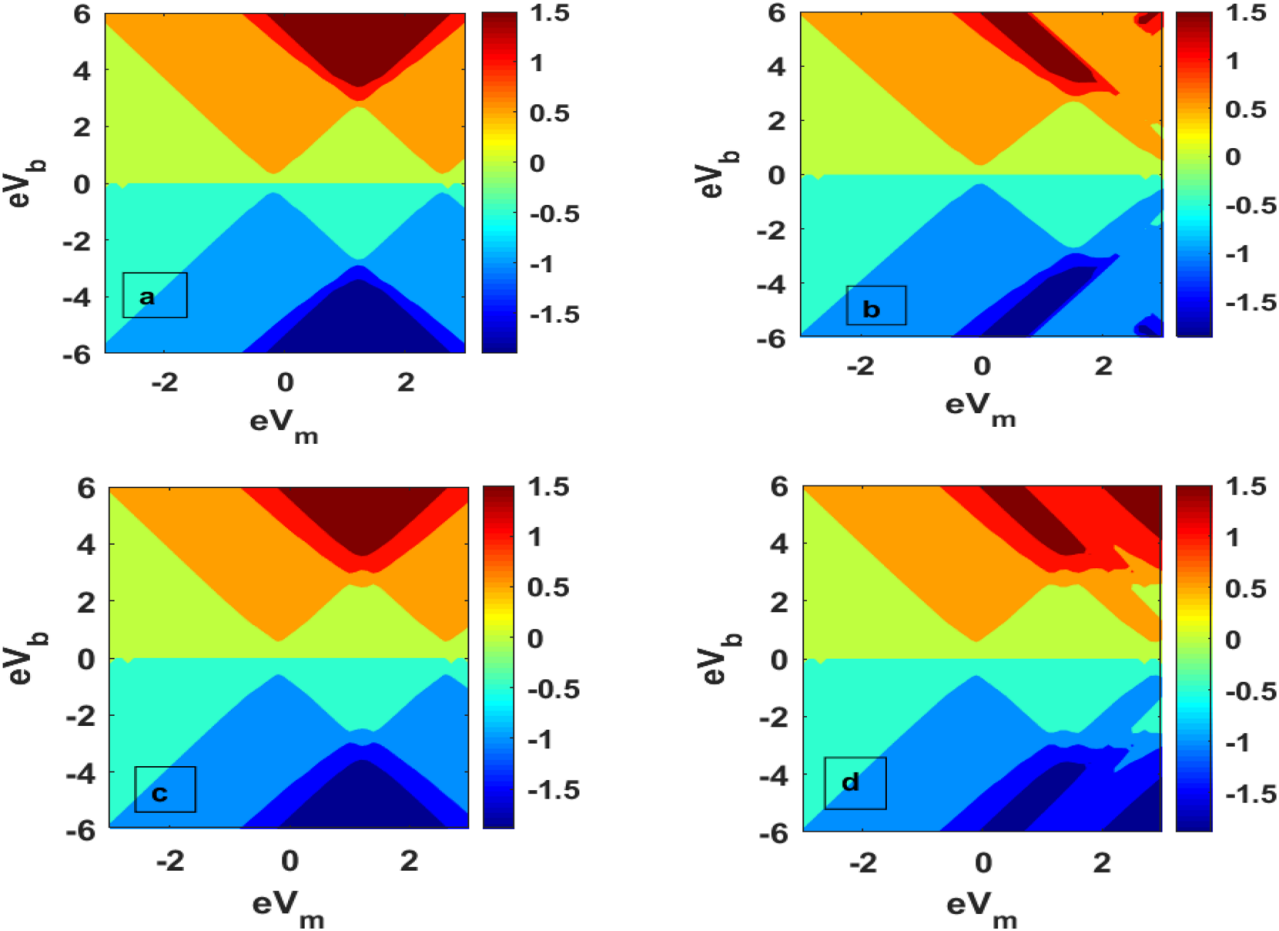
Contour plot of J as a function of *eV*_*m*_ and *eV*_*b*_: (**a**) *μ*_*B*_*B* = 0, *U* = 0; (**b**) *μ*_*B*_*B* = 0, *U* ≠ 0; (**c**) *μ*_*B*_*B* ≠ 0, *U* = 0; (**b**) *μ*_*B*_*B* ≠ 0, *U* ≠ 0.

Figure 6 is a three-dimensional plot of tunneling current for QDD as a function of damping rate and el–ph interaction. In should be noted that the tunneling current shows essentially a Gaussian-like behaviour because of the dominance of the factor $\lambda^{2} e^{{ - \lambda^{2} }}$. The current shows the maximum curvature at λ ≈ 0.5 (Fig. 6a) in the absence of the magnetic field. In presence of a magnectic field, the curvature become small, and *J* has a lower value. Figure 7 gives the contour plots of *J* as a function of the bias voltage and mid voltage. Figure 7a provides the behavour of *J* for $\mu_{B} B = 0,\;U = 0$. Figure 7b shows the behaviour in the presence of el–el interaction. One can see that el–el interaction results in distortion in the current at higher values of the bias voltage and mid voltage. For $\mu_{B} B \ne 0,\;U = 0$, J has a split for higher value of $eV_{m} \;{\text{and}}\;eV_{b}$ (Fig. 7c). When the magnetic field and el–el interaction are present together, both distortion and splitting in the current can be seen at higher value of bias voltage and gate voltage (Fig. 7d).

**Figure 8:**
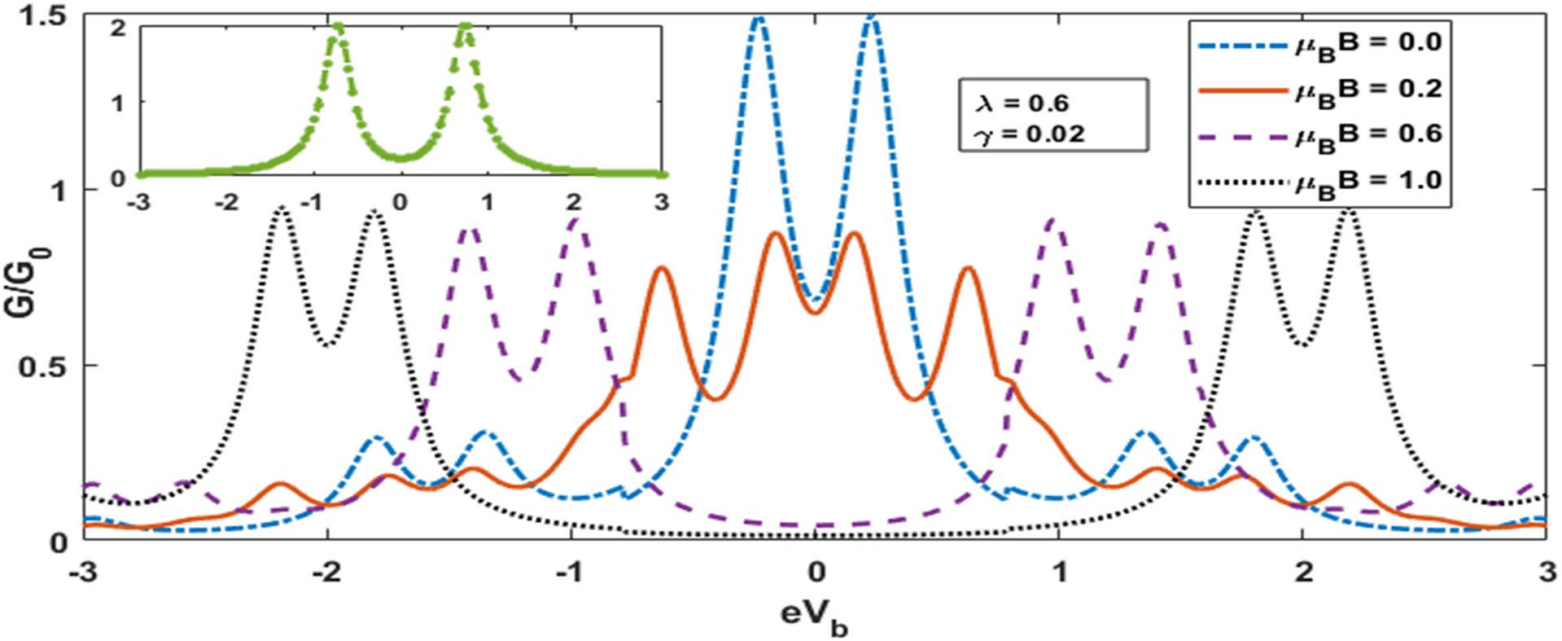
G versus *eV*_*b*_ for different value of *μ*_*B*_*B.*

**Figure 9:**
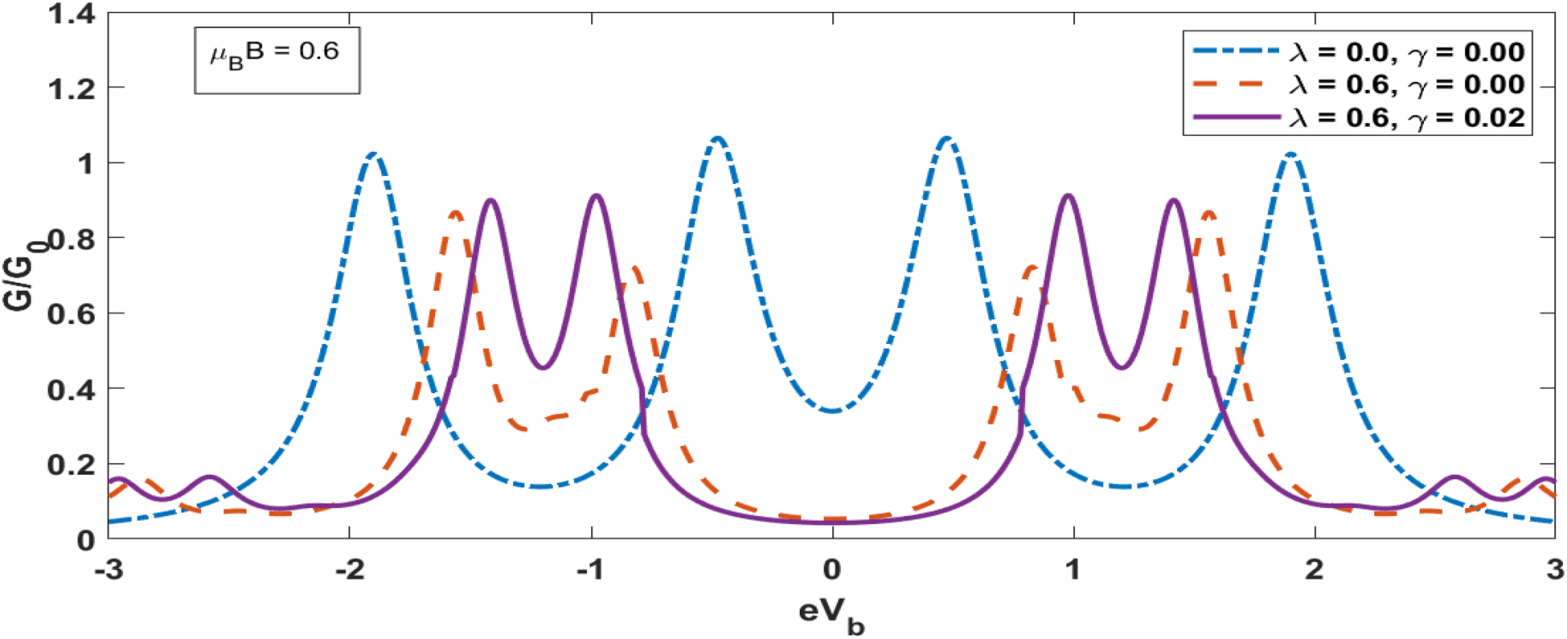
G versus e ${V}_{b}$ for different values of *γ* and *λ.*

Figure 8 shows the plot of Differential Condutance (DC) for QDD as a function of the bias voltage ${\text{e}}V_{b}$ for a few values of the magnetic field. The inset gives the result for DC in the absence of magnetic field, el–ph interaction and the substrare-QDs interaction. In this case, DC has two peaks. These peaks give the possibility of excitation. When the el–ph interaction and damping due to substate are involved but the magnetic field is kept zero, DC decreases and side bands appear along with the lorentzian peaks due to polaronic effect. For a finite value of magnetic field, each peak splits, and show the contribution of spin up and spin down. As Magnetic field increases further we observe sepreation between spin up and down clearly. Also as magnetic field increases the side peaks also shifts further apart. . Figure 9 shows the variation of DC as a function of the bias voltage ${\text{eV}}_{{\text{b}}} .$ In the absence of both el–ph interaction and dissipation, we see a central double-peak structure with two symmetric side peaks. As the el–ph interaction is switched on, the central peaks move away from each other while the earlier side peaks come a little closer and two new small side peaks appear. Thus we do not have any central-peak structure now. The heights of all the peaks however become shorter in heights. As the damping is introduced now, the peak structure becomes more interesting. The peaks rearrange to form two symmetric double-peak structure.

**Figure 10:**
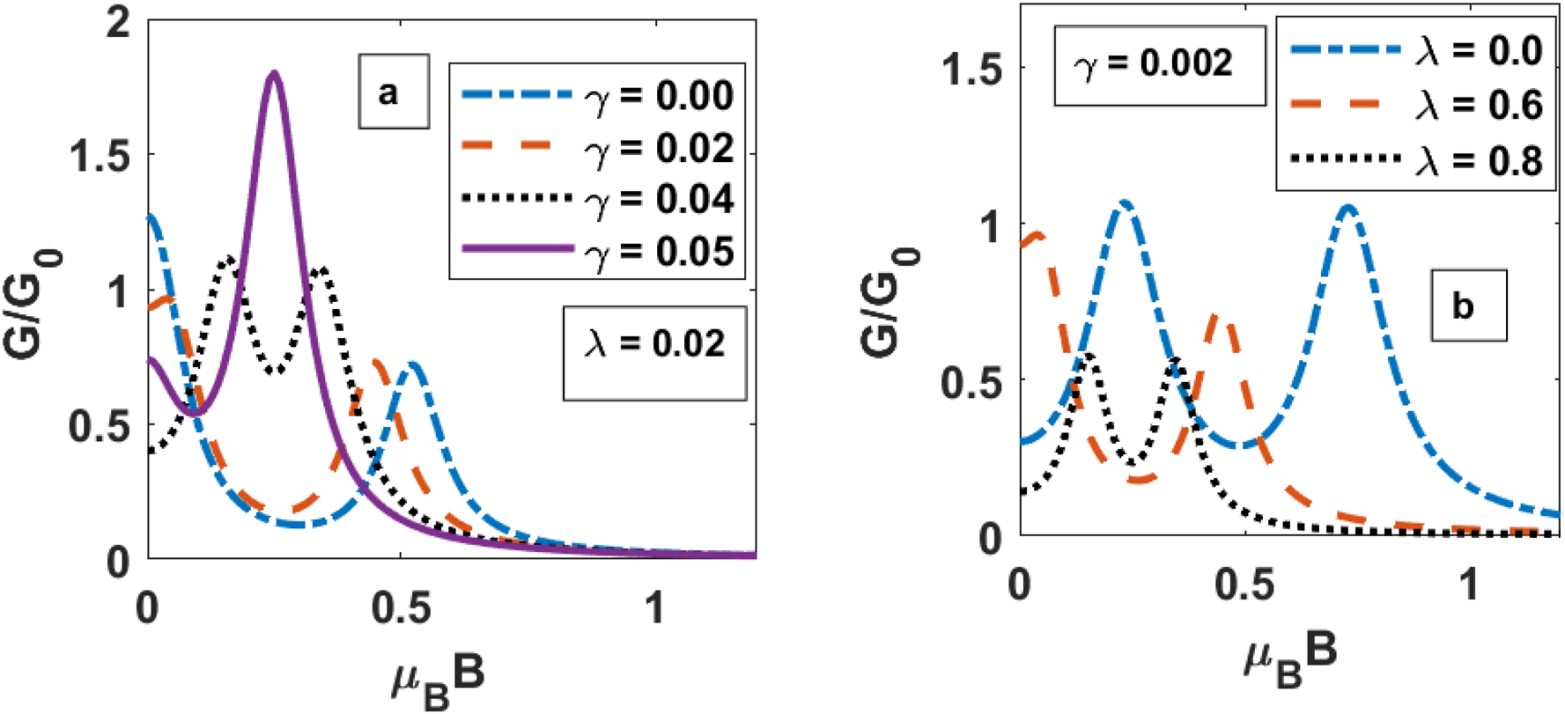
G versus *μ*_*B*_B for different values of λ and γ.

In Fig. 10a, the variation of DC as a function of magnetic field is shown for different values of $\gamma$. When $\gamma = 0$ DC has two unequal peaks. These peaks correspond to spin up and spin down electrons. As the damping is taken into account, the spin-up and spin-down levels shift because of the change in the polaronic effect due to the renormalization of the QD phonon frequency and the peaks of G mentioned above come closer and also the difference between their heights decreases. For $\gamma = 0.05,$ we observe a singlre peak. Similar behaviour is observed with respect to el–ph interaction in the absence of the damping effect in Fig. 10b.

**Figure 11:**
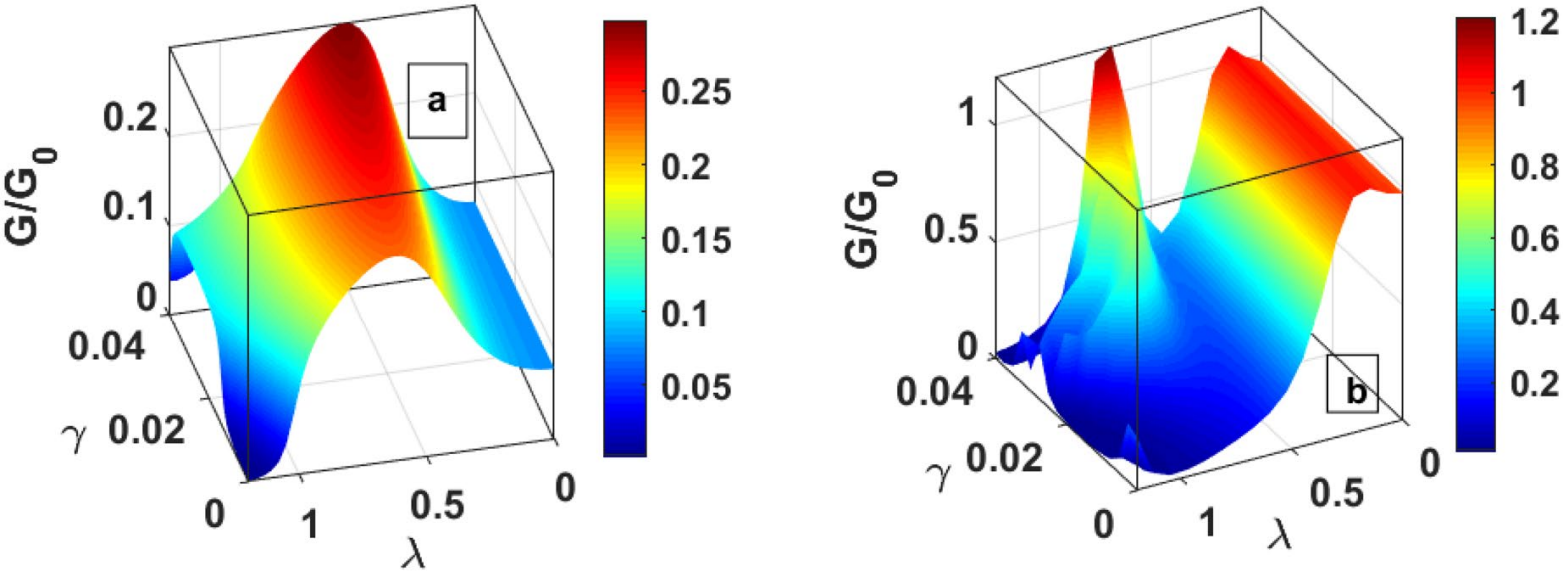
Three-dimentional plot of G as a function of *γ* and λ: (**a**) ${\mu }_{B}B=0$, (**b**) ${\mu }_{B}B\ne 0$.

**Figure 12:**
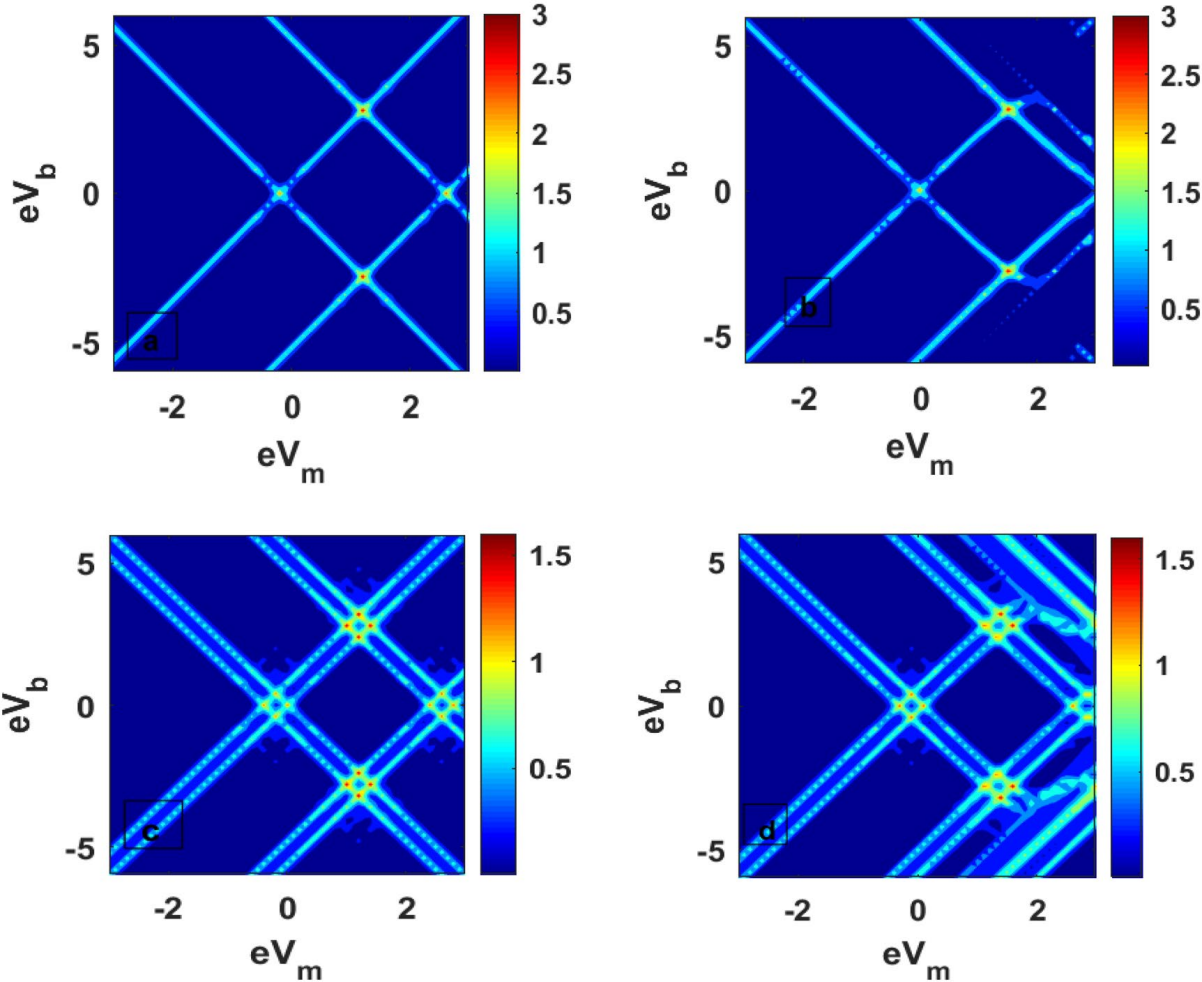
Contour plot G as a function of *eV*_*b*_ and *eV*_*m*_ for (**a**) U = 0, B = 0, (**b**) *U* = 5, *B* = 0 (**c**) *μ*_*B*_*B* ≠ 0, *U* = 0; (**d**) *μ*_*B*_*B* ≠ 0, *U* ≠ 0.

Figure 11 gives three-dimentional plots for DC of QDD as a function of el–ph interaction and dissipation. Figure 11a shows the behaviour for $B = 0$ and Fig. 11b for $B \ne 0$. The behaviour in Fig. 11a resembles the tunneling current behaviour shown in in Fig. 6a. In the presence of a magnetic field, Fig. 11b, two maxima corresponding to the spin-up and spin-down electrons are clearly visible, which were not so clear in Fig. 6b. In Fig. 12, we draw the contour plots of DC in the $(V_{b} - V_{m} \_) -$ plane. Figure 12a is for $U = 0, \;B = 0$, Fig. 12b is for $U \ne 0, \;B = 0$, Fig. 12c is for $U = 0, \;B \ne 0$ and Fig. 12d is for $B \ne 0, \;U \ne 0$. When $U = 0, \;B = 0$, DC is smooth everywhere. In the case of $U \ne 0,\; B = 0,$ we see distotion in G for higher values of mid-voltage and bias voltage. In the case of $U = 0, \;B \ne 0$, we see a split because of the removal of spin degeneracy. Finally, in the case of $B \ne 0,\; U \ne 0,$ one may notice splits together with distortion.

**Figure 13:**
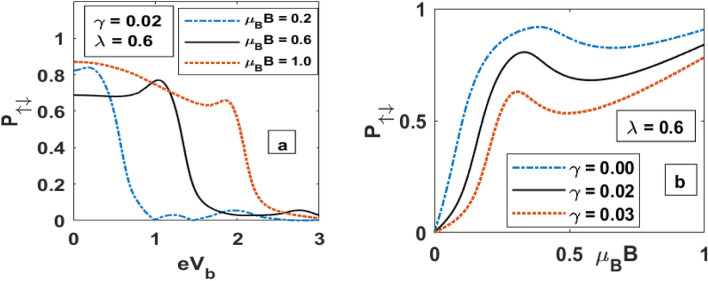
Spin polarization plot for QDD as a function of (**a**) *eV*_*b*_ and (**b**) *μ*_*B*_B.

The Spin polarization as a function of the bias voltage and magnetic field is studied in Fig. 13. Figure 13a shows a decrease in polarization parameter with increasing $eV_{b} .$ As observed in Fig. 4, both the spin-up and spin-down currents increase with the bias voltage, but as the bias voltage increases, the difference in the rate of increase of the two currents decreases leading to a decrease in the polarization parameter. When plotted as a function of the magnetic field, Fig. 13b shows that the spin polarization parameter initially increases with the magnetic field and exhibits two maxima. As damping increases, the spin polarization parameter decreases in general. This happens because damping leads to an upward shift of both the spin-up and spin-down levels, causing a decrease in the spin-up current and an increase in the spin leading to an overall reduction in the polarization parameter. The maximum structure is consistent with Fig. 5.

## Conclusion

In this work, the non-equilibroum transport through a side-coupled QD connected to a source and drain and mounted on a insulating substrate is studied in the presence of el–ph interaction, el–el interaction, magnetic field and dissipation. The system is modeled by the Anderson–Holstein–Caldeira–Leggett Hamiltonian (AHCL) and the Keldysh Green’s function method is employed to calculate the spectral function A, tunneling Current density J, Differential Conductance G and spin polarization parameter $P_{\sigma , - \sigma }$. In the presence of a magnetic field, the spin degeneracy is lifted, leading to the split in the energy levels and the spectral function of QDs. It is shown that an increase in the magnetic field and el–ph interaction reduces the spin-down current $J_{ \downarrow }$ and enhances the spin-up current $J_{ \uparrow }$. The lifting of the spin-degeneracy in presence of a magnetic field also shows up in the G-plot. G also shows a peak structure and height of the peak is found to increase as the magnetic field increases. The spin polarization coefficient of QDD has been calculated as a function of the bias voltage and the magnetic field. We find that as a function of the bias voltage, the spin polarization parameter generally shows a decreasing behaviour except for a shoulder which shifts towards higher voltage as the magnetic field increases. As a function of the magnetic field, the polarization parameter shows in general, an increasing behaviour except for a maximum at some critical value of the magnetic field. This critical field shifts to the left as the dissipation increases. This work can have important applications in nano-devices. For example, the staircase nature of current versus bias voltage which mainly arises due to the second QD is crucial because it provides a precise control on the flow of electrons, which is essential for various electronic and quantum computing applications because it allows to manipulate quantum states and perform quantum operations. This behaviour can also be used in high-precision sensors and single-electron logic circuits. The present work may also have importance in the study of biological clusters where one wishes to explore the properties of a single molecule connected to others. The work can be modified to understand how a cluster of molecules effects the transport properties in presence of interactions and dissipations in a multi-molecular transistor.

## Data Availability

The datasets used and/or analysed during the current study are available from the corresponding author on reasonable request.
